# Editorial: Women in science: Health economics 2022

**DOI:** 10.3389/fpubh.2023.1163995

**Published:** 2023-03-30

**Authors:** Tarang Sharma, Susmita Chatterjee

**Affiliations:** ^1^Evidence to Policy, Hørsholm, Denmark; ^2^Research Department, George Institute for Global Health, New Delhi, India; ^3^Faculty of Medicine, University of New South Wales, Kensington, NSW, Australia; ^4^Prasanna School of Public Health, Manipal Academy of Higher Education, Manipal, India

**Keywords:** women in STEM, health policy, health economics, health financing, women's health

Women are underrepresented in positions of leadership compared to their male counterparts with equivalent experience and skills across different organizations and in academia (1, 2). When we consider the field of scientific research, we know from an analysis of a large-scale administrative dataset on research teams that women are significantly less likely than men to be credited with authorship for articles or patents (3). That is, women accounted for only 34.85% of the authors even though they made up just under half of the workforce (48.25%) in the research teams (3). As in academia and scientific research, authorship plays an integral part in promotions and moving up to management and leadership positions; women lose out as their contributions are not accurately accredited.

In recent years, the COVID-19 pandemic has intensified levels of pre-existing widespread inequalities between women and men; therefore, it is imperative to prioritize policies that enable and encourage women to participate in the labor force (4) and provide a platform for their achievements to be acknowledged. When significant numbers of women entered the field of agricultural economics in the 1980s, they expanded the field which had a narrow focus limited to production, commercial agriculture, and farm management to a wider perspective. Women agricultural economists were interested in addressing societal challenges and, therefore, galvanized the research into including the economics of the environment and natural resources, food safety and nutrition, and global rural and agricultural development, which has benefited the field greatly (5). More women are joining the field of health economics since the 1990s, and the Women in Science: Health Economics from Frontiers helps to promote their work.

This is the second Research Topic in this series, and it aims to improve the visibility of women in the field of Health Economics by promoting health economic research led by them. The first series published in 2021 highlighted five such articles (6), and this research collection has also collated five stellar articles that meet these criteria. This celebrates the work of women health economists and women in science, technology, engineering, and mathematics (STEM).

In the first article of the Research Topic, Dobbins et al. look at the U.S. legal cannabis market and how the levels of tetrahydrocannabinol (THC) in the products and other attributes [type of product, chemovars, or presence of cannabidiol (CBD)] seem to drive prices and product composition in legal cannabis dispensaries. They consider data from the state of California where dispensaries across the state were randomly selected and then screened for a web presence and their product menus analyzed. They found that most of the products offered were highly potent herbal cannabis products (>15% THC) and they were more expensive regardless of product type or chemovar. CBD was not correlated with price. The authors argue that there is a blurring of the distinction between medical and recreational programs and that consumers and patients need to be informed about the higher risk for overconsumption and cannabis use disorder with these high-THC-content products. Efforts and policies should also be put in place for limiting the availability of highly potent THC products.

The second article from Tackie et al. explores the dynamic nexus between economic growth, industrialization, medical technology, and healthcare expenditure in 16 countries in West Africa. They considered data from the World Development Indicators (WDI) from 2000 to 2019 and divided the countries into two panels—low-income (LI) and lower-middle-income (LMI). They noted that by 2030, non-communicable diseases will surpass communicable, maternal, neonatal, and nutritional diseases as the primary cause of death in West Africa due to industrialization, urbanization, population growth, aging population, and increased living standards. Their results demonstrated industrialization as a major determinant of healthcare expenditure in both the LI and LMI panels. While medical technology was confirmed to decrease healthcare expenditure in the LMI panel, it was not significant in the LI panel. The aged population was found to intensify healthcare expenditure in both the LI and LMI panels. The authors, therefore, propose policy considerations to mitigate the negative impact of this nexus and the current trends in the region.

The third article from Calabrò et al. determines the economic impact that included societal perspectives of diabetic macular edema (DME) and the consequences of increased use of dexamethasone implants in the Italian healthcare setting. Usually considered as a second-line treatment, the authors suggest that if their use is increased it would lead to considerable savings in terms of healthcare professionals' time, follow-up, and productivity lost by patients/caregivers, and they estimate these savings could reduce healthcare costs for the management of DME patients in Italy by €2,058,238 in 5 years.

The fourth article by Chang et al. reports the economic evaluation of the non-specific effects (NSE) of the oral polio vaccine (OPV) against under-five mortality and COVID-19 in two different settings. For Guinea-Bissau, a setting with high child mortality, they modeled the impact of three cycles of an annual national immunization day campaign where children get one bi-valent OPV dosage each year for three consecutive years, compared to a hypothetical cohort without this. In India, they considered a susceptible-exposed-infectious-recovered model where OPV was co-administered alongside COVID-19 vaccines with transmission dynamics of COVID-19 set to that of early 2020. The authors found that for child mortality, the headline cost-effectiveness was $650 per child death averted. For COVID-19, assuming OPV had 20% effectiveness, the incremental cost per death averted was $23,000–65,000 if it were administered simultaneously. If the COVID-19 vaccine availability were delayed, the cost per averted death would decrease to $2,600–6,100. Therefore, their economic evaluations suggest that there is potential for OPV to efficiently reduce child mortality in high-mortality environments and argue that it (or other vaccines with similar NSE) can play a role in the fight against COVID-19 in countries facing COVID-19 vaccine delays.

The final article in this Research Topic by Tomaz et al. studies the impact of income inequality on breast cancer mortality in Brazil while considering the socioeconomic status using ecological study data from Brazil in 2020 and the Global Burden of Disease information system from 2017. The authors concluded that income inequality was prone to poor socioeconomic conditions, and therefore have worse conditions of functional health characterized with the highest mortality from breast cancer in the Brazilian federative units in the year 2017. Therefore, they argue that the Human Development Index is important for improving the health of populations as it reflects the quality of health received, access to health services, as well as other positive indicators.

Through this Research Topic, we encourage women researchers to publish more as lead authors in health economics to reduce the prevailing gender gap and pave their way toward leadership in health economics research. Studies have shown that mentoring from senior economics faculty helps to improve the career track of juniors (7). We similarly hope that our Research Topic will also promote junior researchers to come forward and publish as lead authors under the mentorship of senior women leaders.

## Author contributions

TS had prepared the manuscript draft while SC has revised it for important intellectual content. Both authors approved the final version of the manuscript.
